# Supplementary material for: Knockdown of Vacuolar ATPase Subunit G Gene Affects Larval Survival and Impaired Pupation and Adult Emergence in Henosepilachna vigintioctopunctata
Source: Insects. 2021 Oct 14;12(10):935. doi: 10.3390/insects12100935 (PMC8538789; doi:10.3390/insects12100935)
Supplement: Supplementary file 1 [file insects-12-00935-s001.zip › insects-1384335-SI.pdf]

insects

MDPI

Table S1. Primers used in RT-PCR, dsRNA synthesis and qPCR.

Fragment name	Forward primer	Reverse primer
**RT-PCR**		
HvvATPaseG	GCACATTATGATGTTCAAGCTC	AACTGGGGAGGATTGACTTT
**dsRNA synthesis**		
ds*G*	AACTGGCGTTCCCTTTCC	GCAAGTCAAACACAAGGCATT
ds*egfp*	AAGTTCAGCGTGTCCG	CACCTTGATGCCGTTC
**qPCR**		
qHvvATPaseG	GGCAAGTCAAACACAAGGCA	GGCGTTCCCTTTCCTTACGA
qHvRPS18	CGCAATCAAAGGTGTTGGAAG	GCCTAGGGTTGGCCATAATAG
qHvRPL13	AGCATCCTTCGCTCGTTTAG	TTCGACAACCTGCCATTAGG

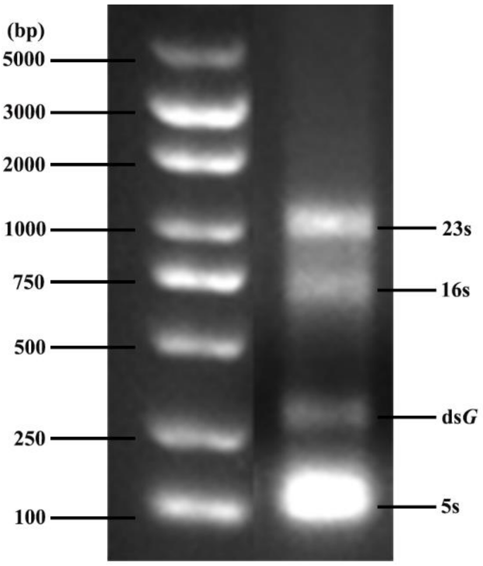

Figure S1. Electrophoresis of target dsRNA on 1% agarose gel.

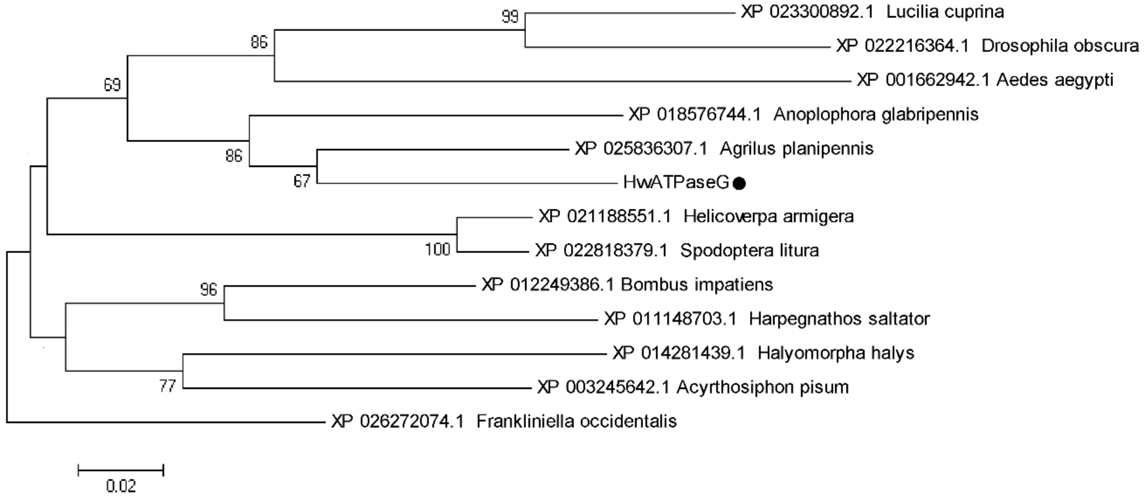

Figure S2. Phylogenetic analysis of vacuolar ATPase V1 subunit G proteins (vATPaseGs). The proteins are derived from three Dipteran *Lucilia cuprina*, *Drosophila melanogaster* and *Aedes aegypti*, three Coleopteran *Henosepilachna vigintioctopunctata*, *Anoplophora glabripennis* and *Agrilus planipennis*, two Lepidopteran *Helicoverpa armigera* and *Spodoptera litura*, two Hymenopteran *Bombus impatiens* and *Harpegnathos saltator*, two Hemipteran *Halyomorpha halys* and *Acyrthopiphon pisum*, and a Thysanopteran *Frankliniella occidentalis*. The tree is constructed using the neighbor-joining method based on the full-length protein sequence alignments. Bootstrap analyses of 1000 replications are carried out and bootstrap values > 50% are shown on the tree.

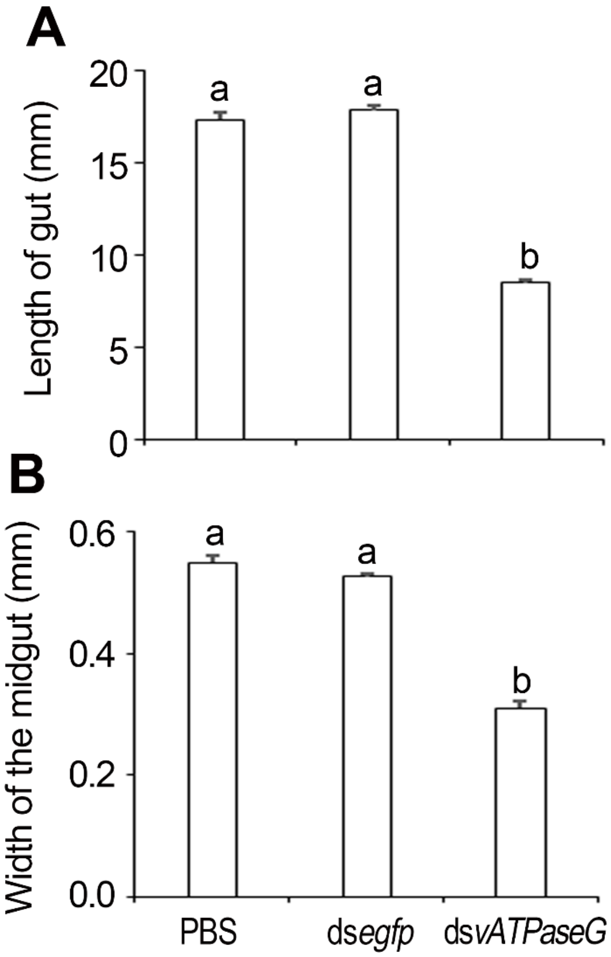

Figure S3. The impacts of dsRNA on the lengths (A) and widths (B) in *Henosepilachna vigintioctopunctata*. The newly-ecdysed fourth instar larvae had ingested PBS-, ds*egfp*-, and ds*vATPaseG*-dipped leaves for three days. The guts were dissected 5 days after the initiation of bioassay. The lengths and widths were measured. The bars represent values (± SE). Different letters indicate significant difference at P value < 0.05 using analysis of variance with the Tukey-Kramer test.

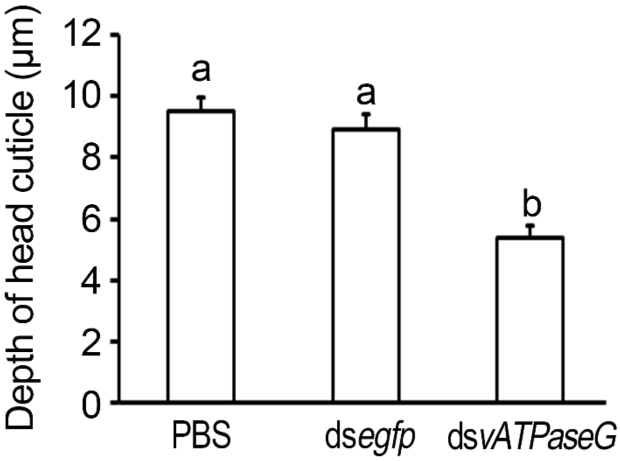

Figure S4. The impacts of dsRNA on the depth of head capsule in *Henosepilachna vigintioctopunctata*. Table 2. days. The sections of the head capsule were stained using hematoxylin-eosin (HE) method. The depths were measured. The bars represent values (± SE). Different letters indicate significant difference at P value < 0.05 using analysis of variance with the Tukey-Kramer test.
